# Common TNF-α, IL-1β, PAI-1, uPA, CD14 and TLR4 polymorphisms are not associated with disease severity or outcome from Gram negative sepsis

**DOI:** 10.1186/1471-2334-7-108

**Published:** 2007-09-18

**Authors:** Kristine Marie Jessen, Sarah Bjerre Lindboe, Anncatrine Luisa Petersen, Jesper Eugen-Olsen, Thomas Benfield

**Affiliations:** 1 Clinical Research Unit, Hvidovre University Hospital, Copenhagen, Denmark; 2 Department of Infectious Diseases, Hvidovre University Hospital, Copenhagen, Denmark

## Abstract

**Background:**

Several studies have investigated single nucleotide polymorphisms (SNPs) in candidate genes associated with sepsis and septic shock with conflicting results. Only few studies have combined the analysis of multiple SNPs in the same population.

**Methods:**

Clinical data and DNA from consecutive adult patients with culture proven Gram negative bacteremia admitted to a Danish hospital between 2000 and 2002. Analysis for commonly described SNPs of tumor necrosis-α, (TNF-α), interleukin-1β (IL-1β), plasminogen activator-1 (PAI-1), urokinase plasminogen activator (uPA), CD14 and toll-like receptor 4 (TLR4) was done.

**Results:**

Of 319 adults, 74% had sepsis, 19% had severe sepsis and 7% were in septic shock. No correlation between severity or outcome of sepsis was observed for the analyzed SNPs of TNF-α, IL-1β, PAI-1, uPA, CD14 or TLR-4. In multivariate Cox proportional hazard regression analysis, increasing age, polymicrobial infection and haemoglobin levels were associated with in-hospital mortality.

**Conclusion:**

We did not find any association between TNF-α, IL-1β, PAI-1, uPA, CD14 and TLR4 polymorphisms and outcome of Gram negative sepsis. Other host factors appear to be more important than the genotypes studied here in determining the severity and outcome of Gram negative sepsis.

## Background

The syndromes of severe sepsis and septic shock are frequent and associated with high mortality [1]. Their pathophysiology is complex and results from the interaction between infecting pathogens and inflammatory and coagulation pathways [2,3]. Among the numerous microorganisms that cause sepsis, Gram negative bacteria, predominantly *Enterobacteriacea*, account for one third of all cases [1].

Innate host defence is integrally linked to inflammation and coagulation [3,4]. Gram negative bacterial lipopolysaccharide (LPS, endotoxin) is sensed by LPS-binding protein (LBP) by the human host. The LPS-LBP complex binds to the cellular surface receptor CD14 and interacts with the toll-like receptor 4 (TLR4) to induce nuclear factor κ-B signalling and transcription of cytokines, chemokines, adhesion and coagulation factors [5]. Among these, tumor necrosis factor α (TNF-α) and interleukin-1β (Il-1β) are decisive proinflammatory mediators. Blood clotting can be initiated by TNF-α and endotoxin and is counteracted by fibrinolysis. Fibrinolysis is initiated by two types of plasminogen activators, the urokinase-type (uPA) and the tissue-type (tPA) and may be inhibited by the plasminogen activator inhibitors, PAI-1 and PAI-2.

Genetic epidemiologic studies suggest a strong genetic influence on the outcome from sepsis [6]. Since dysregulation of innate immunity is believed to be central for the manifestations of sepsis, studies of genetic susceptibility to and outcome of septic shock have focused on genes involved in inflammatory and coagulation pathways. Synonymous and non-synonymous single nucleotide polymorphisms (SNPs) may alter the expression or function of transcribed gene products. We included SNPs that had been shown in other studies to have either clinical or experimental relevance with sepsis outcome through the inflammatory and coagulation pathways. Data indicate that SNPs of TNF-α [7,8], Il-1β [9,10], PAI-1 [11,12], and CD14 [13] may be associated with a poor prognosis from sepsis. Polymorphisms in TLR4 [14] and CD14 [13] are further associated with an increased susceptibility to infection. The uPA polymorphism has not previously been studied in sepsis.

Here we present a genetic association study of Gram negative sepsis with focus on six SNPs previously linked to sepsis pathogenesis and survival.

## Methods

### Patients

All patients older than 17 years admitted to Hvidovre Hospital between June 2000 and May 2002 with a positive blood culture yielding a Gram negative organism were included in the study. Demographic, clinical and laboratory data were extracted on a standardized form. Sepsis, severe sepsis and septic shock were classified according to international guidelines [2]. The study was approved by the Ethics Committee for Copenhagen and Frederiksberg Counties (01-085/2000). None of the patients were lost to follow-up.

### Deoxyribonucleic Acid Extraction

1.5 mL of positive blood culture media was lysed with 1.5 mL of 5 M guanidinium-HCl-100 mM Tris (pH 8.0) [15]. DNA was then extracted with QIAamp mini Spin columns (Qiagen, Hilden, Germany) as described by the manufacturer and stored at -20°C.

### Genotyping

Primers, probes and restriction enzymes are shown in Table 1. The TNF-α SNP was analyzed using a Light Cycler (Roche, Basel, Switzerland) as previously described [16]. Il-1β SNP was analyzed by Polymerase Chain Reaction Restriction Fragment Length Polymorphism (PCR-RFLP) analysis and PAI-1 by allele specific PCR as described [17]. uPA, TLR4, and CD14 were analyzed using a microsphere based assay (Luminex 100, Luminex Corp., Austin, TX). Wild type and mutant allele capture oligonucleotide probes (Table 1) were synthesized and modified at the 5' terminus (TAG Copenhagen, Denmark) and coupled to carboxylated microspheres as described by Luminex. PCR was performed using a multiplex PCR (Qiagen multiplex PCR kit, Qiagen) with biotin-labelled primers (Table 1), and conditions included 95°C for 15 min, 40 cycles of 92°C for 30 seconds, 55°C for 30 seconds and 72°C for 60 seconds. Each biotinylated amplicon was denatured at 95°C for 5 min and hybridized at 54°C for 7.5 min. Samples were filtered through a 1.2 μm Durapore filter, washed, resuspended with streptavidin-R-phycoerythrin, incubated for 10 min and then filtered and washed again before being read with a Luminex-100 according to manufacturer's protocol. All genotyping was performed blinded to study details. The TNF-α, uPA, TLR4, and CD14 SNP results obtained by Lighcycler and Luminex analysis were verified by PCR-RFLP with a minimum of 40 randomly selected samples for each SNP [18-21]. Congruence was found for all samples. IL-1β and PAI-1 SNPs were not assessed by other means.

**Table 1:** Primers, probes and restriction enzymes

**Gene**	**SNP**	**Sense primer**	**Antisense primer**	**Probe/restriction enzyme**
TNF-α	G- 308 A	5'-TAGGTTTTGAGGGGCATGGGGAC-3'	5'- TCTCGGTTTCTTCTCCATCG -3'	5'-TAGGTTTTGAGGGGCATGGGGAC-3'-fluorescein 5'-LC Red640-GGGTTCAGCCTCCAGGGTCCTACACAC-3'-phosphate
Il-1β	C3953T	5'-CACTCCCAGCTTCATCCCTA-3'	5'-AGGTGCATCGTGCACATAAG-3'	*Taq *I
PAI-1	4G/5G	5G allele; 5 ' -GTCTGGACACGTGGGGG-3 ' 4G allele; 5 ' -GTCTGGACACGTGGGGA-3 '	5 ' -TGCAGCCAGCCACGTGATTGTCTAG-3 '	None
uPA	C 422 T	5'-biotin-ACTGCAGGAACCCAGACAAC-3'	5'-AGGGAGGCAGGTAGGAGAAA-3'	5'-GCCTAAAGC**C**GCTTGTCCAA-3' 5'-GGCCTAAAGC**T**GCTTGTCCAA-3'
TLR4	A 896G	5'-biotin-AGTCCATCGTTTGGTTCTGG-3'	5'-AATAGTCACACTCACCAGGGAAA-3'	5'-AGTCAATAATA**T**CATCGAGGTAG-3' 5'-AGTCAATAATA**C**CATCGAGGTAG-3'
CD14	C-159T	5'-biotin-CACCCACCAGAGAAGGCTTA-3'	5'-ATCACCTCCCCACCTCTCTT-3'	5'-GGAGGGGG**G**CCGTAACA-3' 5'-GGGAGGGGG**A**CCGTAACAG-3'

TNF-α: tumor necrosis factor alpha; Il-1β: interleukin-1 beta; PAI-1: plasminogen activator inhibitor 1; uPA: urokinase-type plasminogen activator; TLR4: toll-like receptor 4; CD14: cluster of differentiation 14; A: adenine; T: thymidine; C: cytosine; G: guanine; SNP: single nucleotide polymorphism; LC: light cycler

### Hardy-Weinberg equilibrium (HWE)

HWE analysis was performed for each SNP by comparing the detected genotype distribution with the theoretical distribution estimated on the basis of the SNP allelic frequencies. P > 0.05 (χ^2 ^statistics) was considered to indicate equilibrium.

### Statistics

Genotype distributions were compared using χ^2 ^statistics. Relative risk (RR) with 95% confidence interval (CI) of in-hospital mortality associated with genotypes and other variables was estimated using Cox proportional hazards regression analysis by forced entry. Each covariate was entered separately and covariates that were associated with disease at the P < 0.1 level were included in the multivariate model. Survival curves were constructed by the method of Kaplan-Meier. The date of diagnosis (baseline) was defined as the date of blood culture. Analysis was performed with SPSS 11.5 (Statistical Package for Social Sciences, Chicago, IL.). Power And Precision 2.00 (Biostat, Englewood, NJ) was used to calculate statistical power to detect changes in survival.

## Results

### Patient characteristics

From June 2000 through May 2002, 452 consecutive episodes of Gram negative bacteremia were diagnosed among 427 individuals at Hvidovre Hospital. Of these, 319 were a first episode and had DNA collected. There were no statistical significant differences between individuals included and excluded from the present study. The median age was 76 (interquartile range: 61–84), 172 (54%) of subjects were women, and 255 (80%) had at least one chronic underlying illness. In most patients the infectious focus was the urinary tract (83%) and the most common Gram negative bacteria were *Escherichia coli *(69%) and *Klebsiella pneumoniae *(11%). Nineteen other Gram negative bacteria accounted for the remaining 20%.

### Genotypes

Of 319 included specimens, TNF-α was amplifiable in 304, Il-1β in 317, PAI-1 in 316, uPA in 313, TLR4 in 301, and CD14 in 314. All genotypes were in Hardy-Weinberg equilibrium. Distributions are shown in Table 2.

**Table 2:** Association between genotype and disease severity

**Genotype**	**Sepsis (%)**	**Severe sepsis (%)**	**Septic shock (%)**	**All (%)**	**P value**
**TNF-α **(G-308A)					
GG	134 (60)	35 (58)	13 (65)	182 (60)	
GA	80 (36)	20 (33)	6 (30)	106 (35)	
AA	10 (4)	5 (9)	1 (5)	16 (5)	0.92

**Il-1β **(C3953T)					
CC	135 (57)	32 (53)	11 (52)	178 (56)	
CT	83 (35)	24 (40)	10 (48)	117 (37)	
TT	18 (8)	4 (7)	0	22 (7)	0.5

**PAI-1 **(4G/5G)					
4G-4G	67 (28)	16 (27)	7 (33)	90 (28)	
4G-5G	134 (58)	37 (62)	12 (57)	183 (58)	
5G-5G	35 (14)	7 (11)	2 (10)	44 (14)	0.89

**uPA **(C422T)					
CC	135 (58)	41 (67)	11 (52)	187 (60)	
CT	87 (38)	19 (31)	8 (38)	114 (36)	
TT	9 (4)	1 (2)	2 (10)	12 (4)	0.30

**TLR4 **(A 896G)					
AA	204 (91)	52 (91)	18 (86)	274 (91)	
AG	19 (9)	5 (9)	3 (14)	27 (9)	
GG	0	0	0	0	0.52

**CD14 **(C-159T)					
CC	72 (31)	13 (22)	7 (33)	92 (29)	
CT	117 (50)	30 (51)	9 (43)	156 (50)	
TT	45 (19)	16 (27)	5 (24)	66 (21)	0.57

TNF-α: tumor necrosis factor alpha; Il-1β: interleukin-1 beta; PAI-1: plasminogen activator inhibitor 1; uPA: urokinase-type plasminogen activator; TLR4: toll-like receptor 4; CD14: cluster of differentiation 14; A: adenine; T: thymidine; C: cytosine; G: guanine

### Genotypes and baseline characteristics

No associations were found between genotypes and demographics (age, sex, and comorbidity), temperature, mean arterial blood pressure, white blood cell count, C-reactive protein or causative organism at the time of blood culture.

### Disease severity and genotype

The majority of patients met the criteria for sepsis at the time of blood culture (74%). 19% met the criteria for severe sepsis and 7% were in septic shock.

Neither TNF-α, Il-1β, PAI-1, uPA, TLR4, nor CD14 SNPs were associated with disease severity.

### Mortality and genotype

**Figure 1:**
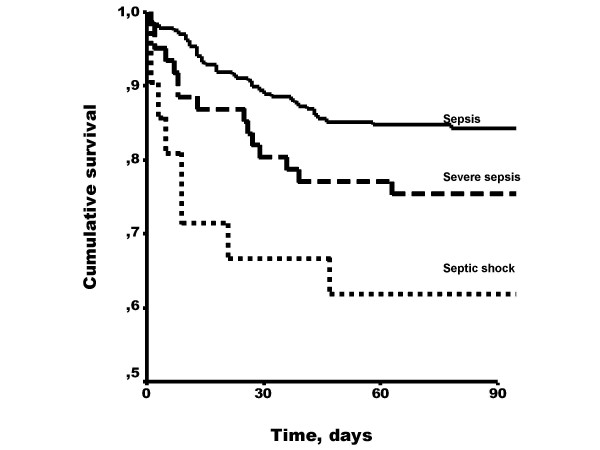
90-day survival from Gram negative sepsis according to disease severity. Log-rank test: P = 0.014.

In total 63 (19%) died during hospitalization. Mortality increased with severity of disease from 17% for sepsis to 25% and 38% for severe sepsis and septic shock, respectively (Figure 1). We did not detect any association between the tested SNPs and outcome (in-hospital, 1 or 3 month mortality rates) when tested for the whole group or tested for each of the severity groups (Table 3).

**Table 3:** In-hospital mortality according to genotype

**Genotype**	**In-hospital mortality (%)**	**P value**
**TNF-α **(G-308A)		
GG	34/182 (19)	
GA	20/106 (19)	
AA	6/16 (38)	0.19

**Il-1β **(C3953T)		
CC	32/178 (18)	
CT	25/117 (21)	
TT	5/22 (23)	0.61

**PAI-1 **(4G/5G)		
4G-4G	15/90 (17)	
4G-5G	37/183 (20)	
5G-5G	11/44 (25)	0.61

**uPA **(C422T)		
CC	40/187 (21)	
CT	20/114 (18)	
TT	2/12 (17)	0.86

**TLR4 **(A 896G)		
AA	49/274 (18)	
AG	8/27 (30)	
GG	-	0.11

**CD14 **(C-159T)		
CC	18/92 (20)	
CT	33/156 (21)	
TT	11/66 (17)	0.90

TNF-α: tumor necrosis factor alpha; Il-1β: interleukin-1 beta; PAI-1: plasminogen activator inhibitor 1; uPA: urokinase-type plasminogen activator; TLR4: toll-like receptor 4; CD14: cluster of differentiation 14; A: adenine; T: thymidine; C: cytosine; G: guanine

### Multivariate analysis of factors associated with outcome

A possible association of genotypes and other baseline variables with in-hospital mortality was further explored by Cox regression analysis. By univariate analysis, increasing age, disease severity, polymicrobial infection, intensive care admission, lower haemoglobin, leukocytosis, elevated C-reactive protein, elevated plasma urea, and elevated alanine transferase was associated with in-hospital mortality. Sex, comorbidity, alcoholism, mean arterial pressure, temperature, lymphocyte count, platelet count, bilirubin and lactate dehydrogenase was not associated with in-hospital mortality. Variables were then grouped in two categories due to the many covariates associated with mortality in univariate analysis relative to the number of events and, thereby, the risk of overfitting the multivariate analysis. Model 1 consisted of demographic variables and microbiological findings and model 2 consisted of laboratory values. Variables associated with in-hospital mortality at P < 0.05 were then fitted in a third model. By this approach, increasing age and polymicrobial infection was associated with a poor outcome, whereas increasing haemoglobin levels were associated with a better outcome (Table 4). Finally, based on this analysis, we stratified for polymicrobial infection and/or entered age and/or haemoglobin levels in the Cox analysis in order to investigate whether SNPs affected outcome in subgroups of patients with Gram negative sepsis. None of the SNPs were associated with outcome.

**Table 4:** Multivariate analysis of factors associated with in-hospital mortality

	**Model 1**		**Model 2**		**Model 3**	
**Variable**	RR (95% CI)	P value	RR (95% CI)	P value	RR (95% CI)	P value

**Age, per year increment**	1.02 (1.00–1.04)	0.026	-		1.02 (1.00–1.03)	0.053
**Diseases severity**			-		-	
Sepsis	1.0					
Severe sepsis	1.25 (0.65–2.39)					
Septic shock	1.70 (0.48–6.05)	0.674				
**Intensive care admission**			-		-	
No	1.0					
Yes	2.50 (0.85–7.42)	0.098				
**Polymicrobial infection**			-			
No	1.0				1.0	
Yes	3.02 (1.58–7.41)	0.001			2.52 (1.27–5.01)	0.008
**Hemoglobin, per μmol/L increase**	-		0.71 (0.52–0.97)	0.031	0.70 (0.56–0.88)	0.002
**Leukocyte count, per 10**^9^**/mL increase**	-		1.00 (0.96–1.05)	0.795	-	
**C-reactive protein, per milligram/L increase**	-		1.00 (0.99–1.00)	0.287	-	
**Urea, per μmol/L increase**	-		1.02 (0.98–1.06)	0.234	-	
**Alanine transferase, per unit/L increase**	-		0.99 (0.98–1.00)	0.195	-	

RR: relative risk; CI: confidence interval

## Discussion

We did not find any association between disease severity and putative SNPs involved in inflammatory and coagulation pathways during the course of sepsis nor were any of the SNPs associated with outcome. The homozygous TNF-α AA and heterozygous TLR4 AG SNPs were associated with an increased but statistically non-significant risk of death.

Genetic association studies notoriously contradict one another [22]. Conflicting results have been reported for TNF-α (G-308A), which was associated to disease severity and outcome in some [7,8,23] but not other studies [24-26]. Controversy also exists regarding the role of for PAI-1 [11,12,27], CD14 (C-159T) [13,28], TLR4 (A896G) [20,29], although IL-1β (C3953T) have consistently been reported to be without association to disease severity or outcome [30]. Methological problems may explain many of the discrepancies. In particular, many studies have insufficient sample size to make firm statistical conclusions. The present study provided > 80% power to detect a 1.5-fold increase in relative risk of death for the heterozygous forms of TNF-α, Il-1β, PAI-1, uPA, and CD14, while a 2.5-fold was necessary to provide 80% power to detect differences associated with the lesser frequent allele frequencies of TLR4. Although, the level of association may be debated, we find 1.5–2.5-fold changes reasonable. The study was, however, underpowered to detect associations with the lesser frequent homozygous TNF-α, IL-1β, uPA and heterozygous TLR4 genotypes. Publication bias may also influence the true genetic association between disease severity and outcome because negative association studies are less likely to be published than studies that find an association. Our negative findings may be due to the fact that our population differs from the previous reported studies showing a positive correlation, e.g. studies of meningococcal disease alone (PAI-1, TLR4 and TNF-α) and of patients with severe sepsis in intensive care settings (TNF-α and TLR4).

Strengths of the present study include the fact that the cohort represents unselected and consecutive patients with Gram negative sepsis. Limitations relate to the sample size because larger samples would detect smaller associations than studied here. Meta-analysis of multiple cohorts could add power to discriminate between potential genetic associations and statistical uncertainties of polymorphisms of genes in the inflammatory and coagulative pathways. Use of novel technologies that permit genome-wide genetic analysis of SNPs and haplotypes will likely be useful in future genetic association studies.

## Competing interests

The author(s) declare that they have no competing interests.

## Authors' contributions

KMJ, SBL, and ALP developed and performed the genetic analysis. JEO and TB were responsible for conception, design, data collection and statistical analysis. All authors participated in the writing of the manuscript. All authors have read and approved the final version of the manuscript.

## Pre-publication history

The pre-publication history for this paper can be accessed here:



## References

[B1] Martin GS, Mannino GM, Eaton S, Moss M (2003). The epidemiology of sepsis in the United States from 1979 through 2000.

[B2] Bone RC, Balk RA, Cerra FB, Dellinger RP, Fein AM, Knaus WA, Schein RM, Sibbald WJ (1992). Definitions for sepsis and organ failure and guidelines for the use of innovative therapies in sepsis. The ACCP/SCCM Consensus Conference Committee. American College of Chest Physicians/Society of Critical Care Medicine. Chest.

[B3] Russell JA (2006). Management of sepsis. N Engl J Med.

[B4] Esmon CT (2004). Interactions between the innate immune and blood coagulation systems. Trends Immunol.

[B5] Beutler B, Hoebe K, Du X, Ulevitch RJ (2003). How we detect microbes and respond to them: the Toll-like receptors and their transducers. J Leukoc Biol.

[B6] Sorensen TI, Nielsen GG, Andersen PK, Teasdale TW (1988). Genetic and environmental influences on premature death in adult adoptees. N Engl J Med.

[B7] Nadel S, Newport MJ, Booy R, Levin M (1996). Variation in the tumor necrosis factor-a gene promoter region may be associated with death from meningococcal disease.. J Infect Dis.

[B8] Mira JP, Cariou A, Grall F, Delclaux C, Losser MR, Heshmati F, Cheval C, Monchi M, Teboul JL, Riche F, Leleu G, Arbibe L, Mignon A, Delpech M, Dhainaut JF (1999). Association of TNF2, a TNF-a promoter polymorphism, with septic shock susceptibility and mortality.. JAMA.

[B9] Pociot F, Molvig J, Wogensen L, Worsaae H, Nerup J (1992). A TaqI polymorphism in the human interleukin-1 beta (IL-1 beta) gene correlates with IL-1 beta secretion in vitro. Eur J Clin Invest.

[B10] Walley AJ, Aucan C, Kwiatkowski D, Hill AV (2004). Interleukin-1 gene cluster polymorphisms and susceptibility to clinical malaria in a Gambian case-control study. Eur J Hum Genet.

[B11] Hermans PW, Hibberd ML, Booy R, Daramola O, Hazelzet JA, de Groot R, Levin M (1999). 4G/5G promoter polymorphism in the plasminogen-activator-inhibitor-1 gene and outcome of meningococcal disease. Meningococcal Research Group.. Lancet.

[B12] Westendorp RG, Hottenga JJ, Slagboom PE (1999). Variation in plasminogen-activator-inhibitor-1 gene and risk of meningococcal septic shock.. Lancet.

[B13] Gibot S, Cariou A, Drouet L, Rossignol M, Ripoll L (2002). Association between a genomic polymorphism within the CD14 locus and septic shock susceptibility and mortality rate. Crit Care Med.

[B14] Smirnova I, Mann N, Dols A, Derkx HH, Hibberd ML, Levin M, Beutler B (2003). Assay of locus-specific genetic load implicates rare Toll-like receptor 4 mutations in meningococcal susceptibility. Proc Natl Acad Sci U S A.

[B15] Fredricks DN, Relman DA (1998). Improved amplification of microbial DNA from blood cultures by removal of the PCR inhibitor sodium polyanetholesulfonate.. J Clin Microbiol.

[B16] Bruunsgaard H, Benfield TL, Andersen-Ranberg K, Hjelmborg Jv JB, Pedersen AN, Schroll M, Pedersen BK, Jeune B (2004). The Tumor Necrosis Factor Alpha -308G>A Polymorphism Is Associated with Dementia in the Oldest Old. J Am Geriatr Soc.

[B17] Margaglione M, Grandone E, Cappucci G, Colaizzo D, Giuliani N, Vecchione G, d'Addedda M, Di MG (1997). An alternative method for PAI-1 promoter polymorphism (4G/5G) typing. Thromb Haemost.

[B18] Wilson AG, di Giovine FS, Blakemore AIF, Duff GW (1992). Single base polymorphism in the human tumour necrosis factor a (TNFa) gene detectable by NcoI restriction of PCR product.. Hum Mol Genet.

[B19] Baldini M, Lohman IC, Halonen M, Erickson RP, Holt PG, Martinez FD (1999). A Polymorphism* in the 5' flanking region of the CD14 gene is associated with circulating soluble CD14 levels and with total serum immunoglobulin E. Am J Respir Cell Mol Biol.

[B20] Lorenz E, Mira JP, Frees KL, Schwartz DA (2002). Relevance of mutations in the TLR4 receptor in patients with gram-negative septic shock. Arch Intern Med.

[B21] Beqaj SH, Post D, Ryan JM (2003). Single-nucleotide polymorphism of the urokinase-plasminogen activator gene during aging and transformation of human diploid kidney cell cultures. In Vitro Cell Dev Biol Anim.

[B22] Clark MF, Baudouin SV (2006). A systematic review of the quality of genetic association studies in human sepsis. Intensive Care Med.

[B23] Tang GJ, Huang SL, Yien HW, Chen WS, Chi CW, Wu CW, Lui WY, Chiu JH, Lee TY (2000). Tumor necrosis factor gene polymorphism and septic shock in surgical infection. Crit Care Med.

[B24] Stuber F, Udalova IA, Book M, Drutskaya LN, Kuprash DV, Turetskaya RL, Schade FU, Nedospasov SA (1995). -308 tumor necrosis factor (TNF) polymorphism is not associated with survival in severe sepsis and is unrelated to lipopolysaccharide inducibility of the human TNF promoter. J Inflamm.

[B25] Reid CL, Perrey C, Pravica V, Hutchinson IV, Campbell IT (2002). Genetic variation in proinflammatory and anti-inflammatory cytokine production in multiple organ dysfunction syndrome. Crit Care Med.

[B26] Gordon AC, Lagan AL, Aganna E, Cheung L, Peters CJ, McDermott MF, Millo JL, Welsh KI, Holloway P, Hitman GA, Piper RD, Garrard CS, Hinds CJ (2004). TNF and TNFR polymorphisms in severe sepsis and septic shock: a prospective multicentre study. Genes Immun.

[B27] Haralambous E, Hibberd ML, Hermans PW, Ninis N, Nadel S, Levin M (2003). Role of functional plasminogen-activator-inhibitor-1 4G/5G promoter polymorphism in susceptibility, severity, and outcome of meningococcal disease in Caucasian children. Crit Care Med.

[B28] Hubacek JA, Stuber F, Frohlich D, Book M, Wetegrove S, Rothe G, Schmitz G (2000). The common functional C(-159)T polymorphism within the promoter region of the lipopolysaccharide receptor CD14 is not associated with sepsis development or mortality. Genes Immun.

[B29] Read RC, Pullin J, Gregory S, Borrow R, Kaczmarski EB, di Giovine FS, Dower SK, Cannings C, Wilson AG (2001). A functional polymorphism of toll-like receptor 4 is not associated with likelihood or severity of meningococcal disease. J Infect Dis.

[B30] Fang XM, Schroder S, Hoeft A, Stuber F (1999). Comparison of two polymorphisms of the interleukin-1 gene family: interleukin-1 receptor antagonist polymorphism contributes to susceptibility to severe sepsis. Crit Care Med.

